# Tertiary Syphilis Masquerading as Oropharyngeal Cancer

**DOI:** 10.7759/cureus.28912

**Published:** 2022-09-07

**Authors:** Caroline R Christmann, Wesley D Figg, Ritodhi Chatterjee, Philip F Lavere, Niraj Mehta

**Affiliations:** 1 Department of Internal Medicine, Baylor College of Medicine, Houston, USA

**Keywords:** immuno suppresion, oral syphilis, hiv aids, oral mucosal lesions, tertiary syphilis, syphilis

## Abstract

Syphilis is re-emerging in the United States. *Treponema pallidum*, the spirochete bacterium responsible for syphilis, has immunoevasive properties that facilitate pathogenesis and widespread tissue involvement. Host immune status, particularly the presence of HIV/AIDS, can influence the presentation and severity of the disease. Patients co-infected with HIV and syphilis may develop atypical lesions, including those involving the oropharynx. Any immunocompromised patient with tongue lesions and lymphadenopathy is presumed to have a wide differential diagnosis, and tissue sampling with histopathologic analysis is indicated. We present a patient with gumma of the tongue as the initial manifestation of tertiary syphilis.

## Introduction

The rate of syphilis tripled between 2013 and 2018 in the United States [1]. The causative spirochete bacterium *Treponema pallidum* widely disseminates in host tissues and is immunoevasive, which taken together, can yield atypical presentations that mimic other diseases years after primary infection [2]. There are a variety of oral lesions associated with syphilis, including a primary painless oral chancre; snail-track ulcerations or lues maligna of the tongue; or gummas characteristic of tertiary syphilis involving the hard palate or, less commonly, the tongue [3]. Lymphadenopathy and local ulceration associated with oral lesions often raise suspicion for head and neck cancer [4]. 

Syphilis co-infection with HIV is common; it facilitates HIV transmission, reduces immune recovery, and impairs antiretroviral efficacy [5,6]. The initial presentation of syphilis is similar regardless of HIV status, though HIV-positive patients are more likely to have deeper and multiple chancres [5]. Here we present an unusual case of tertiary syphilis presenting with an isolated tongue lesion and cervical lymphadenopathy in a patient with HIV/AIDS. 

## Case presentation

**Figure 1:**
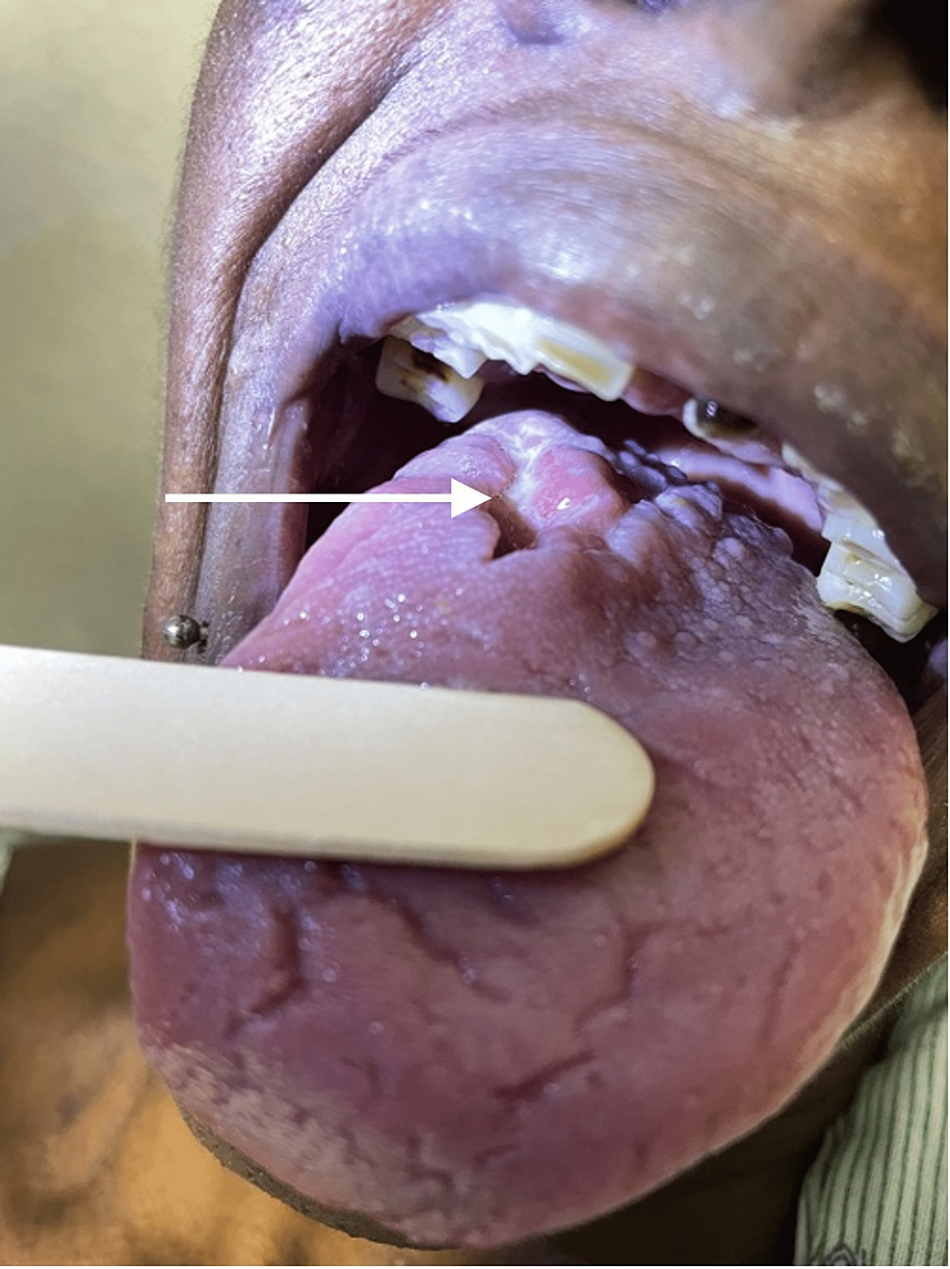
Right posterolateral necrotic tongue lesion

**Figure 2:**
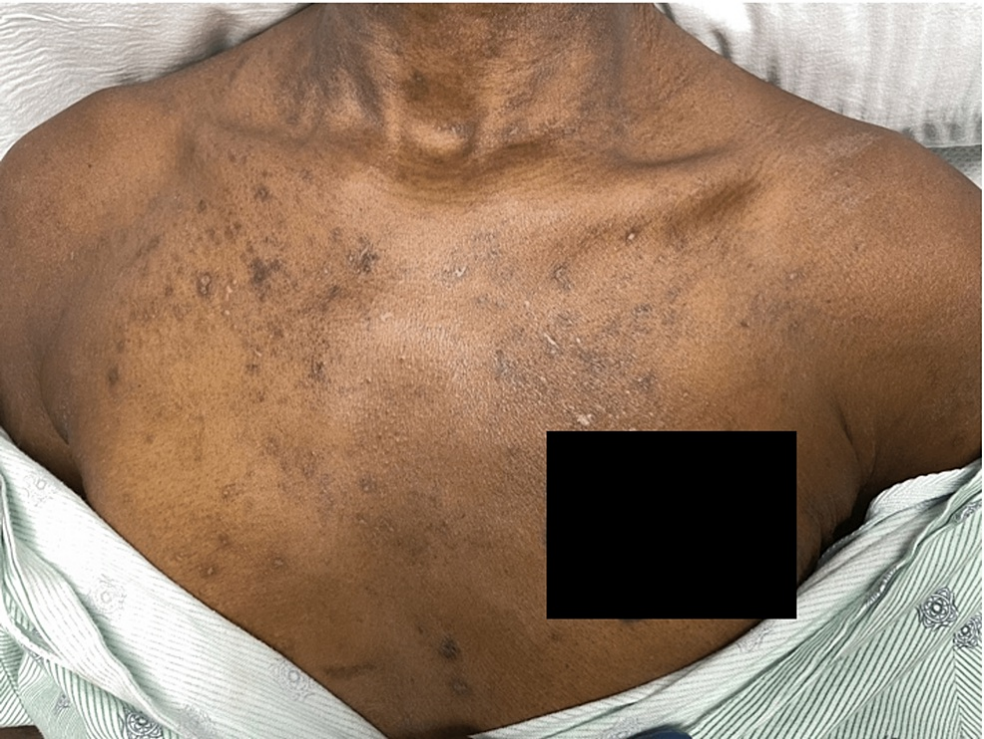
Healing maculopapular rash of the upper chest

**Figure 3:**
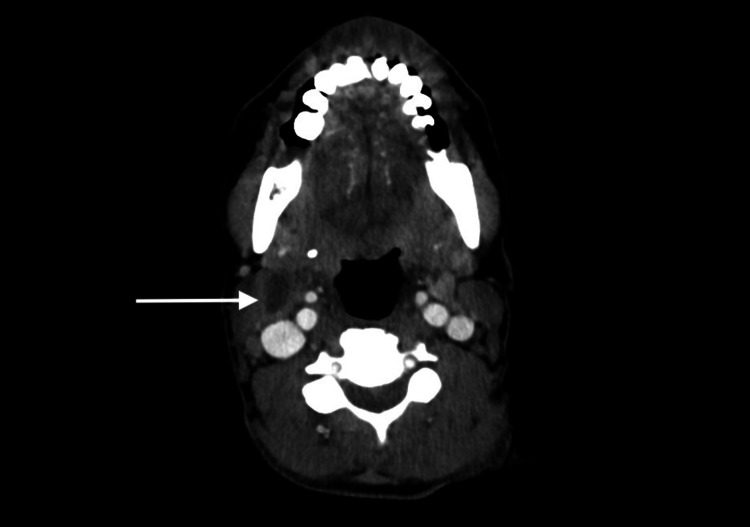
CT scan demonstrating necrotic level IIA lymph node

A 44-year-old female with a 20-year history of HIV off antiretroviral therapy (ART) and a 30-year smoking history experienced 40 lb unintentional weight loss over the past three months. She then presented to the ER with one week of trismus, mouth pain, dysphagia, odynophagia, and decreased oral intake. Physical exam was notable for an ulcerated right posterolateral tongue mass (Figure 1) and maculopapular rash on the upper chest (Figure 2). CD4 (cluster of differentiation 4) count was 142 cells/µL and serum rapid plasma reagin (RPR) 1:256. Health department records revealed remote RPR of 1:2 with no history of treatment. The patient was previously on elvitegravir-cobicistat-emtricitabine-tenofovir (Stribild, Gilead Sciences, Foster City, USA) for ART, last taken one year ago. Computed tomography (CT) scan of the neck without contrast demonstrated a 4.5 cm necrotic tongue lesion as well as a necrotic level IIA lymph node (Figure 3). Lumbar puncture showed lymphocytic pleocytosis, but broad infectious workup was otherwise unremarkable. 

Given the patient’s smoking history and immunocompromised status, the leading diagnosis was tongue-base squamous cell carcinoma (SCC) with nodal extension. Abscess with regional lymphadenitis, or other infections-including fungal, mycobacterial, and treponemal etiologies-were also considered. IV ampicillin-sulbactam 1.5 g every 6 hours was initiated and a biopsy of the tongue lesion via direct laryngoscopy and lymph node via fine needle aspiration was performed. 

Histopathology revealed squamous mucosa and intense lymphoplasmacytic infiltrate without high-grade dysplasia or invasive carcinoma. Acid fast, fungal cultures, herpes simplex virus (HSV), cytomegalovirus (CMV), and human herpes virus (HHV) testing were negative. Send-out specimens were positive for spirochetes on immunohistochemistry staining. Next-generation sequencing was negative for fungal, mycobacterial, non-mycobacterial, and other bacterial organisms. The patient received 2.4 million units of intramuscular benzathine penicillin, the first of three total doses for presumed late-latent syphilis, and was discharged with otolaryngology and infectious disease follow-up. 

## Discussion

Tongue gumma as the initial manifestation of tertiary syphilis is a rare presentation of an increasingly common disease. Oropharyngeal syphilitic lesions encompass a wide range of morphologies, from non-specific mucosal ulceration, formation of pseudomembranes, blistering mucositis, gummas, and tongue-predominant lesions of focal epithelial and fibrous hyperplasia, lymphoid hyperplasia, hyperkeratosis, and necrosis [7,8]. A recent case series reported six patients with oropharyngeal syphilis who were initially diagnosed with SCC, which mirrors our initial hypothesis in the setting of significant weight loss, immunodeficiency, and tobacco use [4]. Histology in this case demonstrated lymphoplasmacytic infiltrate, which has also been reported in oral syphilis [9].

Tertiary syphilis is extremely rare in the antibiotic era. Tertiary syphilis in isolation, without symptoms of primary or secondary syphilis, typically presents with nodular or gummatous lesions [10]. These may present as a variety of clustered plaques, papules, or nodules, often with ulceration [11]. These presentations are easily mistaken for a variety of diseases, including discoid lupus erythematosus, atypical mycobacterial infections, sarcoidosis, and pyoderma gangrenosum [11]. Isolated oral lesions in tertiary syphilis are most commonly ulcerative or destructive lesions on the tongue, as seen in this patient, or on the palate, possibly leading to diffuse atrophy of the tongue or interstitial glossitis [8].

This patient’s HIV-positive status further complicated diagnosis and treatment. Co-infection yields greater infectivity, lower CD4 counts, increasing viral load, earlier progression of syphilis, higher rates of tertiary syphilis, and increased neurologic involvement [1,12-14]. Syphilis co-infection also appears to adversely affect the treatment of HIV, and vice versa [6,15,16]. Compared to ART-naïve HIV-positive patients without syphilis, co-infection is associated with poorer recovery of CD4­+ T cells as well as a greater risk of virologic failure with ART [6]. Although treatment for syphilis is identical regardless of HIV status, HIV positivity may be associated with higher rates of serological failure, and treating syphilis in HIV-positive patients is often associated with therapeutic uncertainty due to slower serologic response rates [15,16]. The synergistic relationship underscores the importance of routine co-testing in individuals with HIV or seropositive RPR titers, and outpatient follow-up for co-infected patients is essential. 

## Conclusions

Rates of syphilis infection are rising, and immunocompromised patients often manifest more severe and atypical presentations. Patients exhibiting grossly non-specific oropharyngeal lesions concerning for malignancy should be screened for syphilis, given the potential for involvement of the oral cavity at any stage. Isolated tertiary syphilis may present with nonspecific gummatous or nodular lesions that cause local destruction and ulceration. Frequent RPR monitoring in high-risk patients and tissue sampling of suspicious lesions can facilitate early diagnosis and expeditious treatment, which can prevent progression into tissue-destructive gummas.

## References

[1] Ren M, Dashwood T, Walmsley S (2021). The Intersection of HIV and syphilis: update on the key considerations in testing and management. Curr HIV/AIDS Rep.

[2] Peeling RW, Mabey D, Kamb ML, Chen XS, Radolf JD, Benzaken AS (2017). Syphilis. Nat Rev Dis Primers.

[3] Jones L, Ong EL, Okpokam A, Sloan P, Macleod I, Staines KS (2012). Three cases of oral syphilis - an overview. Br Dent J.

[4] Jategaonkar A, Klimczak J, Agarwal J, Badhey A, Portnoy WM, Damiano A, Chai RL (2019). Syphilis of the oropharynx: case series of "The Great Masquerader". Am J Otolaryngol.

[5] Zetola NM, Klausner JD (2007). Syphilis and HIV infection: an update. Clin Infect Dis.

[6] Fan L, Yu A, Zhang D, Wang Z, Ma P (2021). Consequences of HIV/syphilis co-infection on HIV viral load and immune response to antiretroviral therapy. Infect Drug Resist.

[7] Leuci S, Martina S, Adamo D (2013). Oral Syphilis: a retrospective analysis of 12 cases and a review of the literature. Oral Dis.

[8] Smith MH, Vargo RJ, Bilodeau EA (2021). Oral manifestations of syphilis: a review of the clinical and histopathologic characteristics of a reemerging entity with report of 19 new cases. Head Neck Pathol.

[9] Barrett AW, Villarroel Dorrego M, Hodgson TA, Porter SR, Hopper C, Argiriadou AS, Speight PM (2004). The histopathology of syphilis of the oral mucosa. J Oral Pathol Med.

[10] Revathi TN, Bhat S, Asha GS (2011). Benign nodular tertiary syphilis: a rare presenting manifestation of HIV infection. Dermatol Online J.

[11] Pereira TM, Fernandes JC, Vieira AP, Basto AS (2007). Tertiary syphilis. Int J Dermatol.

[12] Buchacz K, Patel P, Taylor M, Kerndt PR, Byers RH, Holmberg SD, Klausner JD (2004). Syphilis increases HIV viral load and decreases CD4 cell counts in HIV-infected patients with new syphilis infections. AIDS.

[13] Arora PN, Sastry CV (1992). HIV infection and genital ulcer disease. Indian J Sex Transm Dis.

[14] Flores JL (1995). Syphilis. A tale of twisted treponemes. West J Med.

[15] Ghanem KG, Erbelding EJ, Wiener ZS, Rompalo AM (2007). Serological response to syphilis treatment in HIV-positive and HIV-negative patients attending sexually transmitted diseases clinics. Sex Transm Infect.

[16] Clement ME, Okeke NL, Hicks CB (2014). Treatment of syphilis: a systematic review. JAMA.

